# Biomarkers and clinical rules for the management of mild traumatic brain injury: a narrative review

**DOI:** 10.1186/s12245-025-01088-8

**Published:** 2026-01-24

**Authors:** Sebastián Salgado, Vicente Saver, Ángel Sáenz, Andrés Ferre, Andrés Giglio, Andrés Reccius

**Affiliations:** 1 Critical Care Unit, Clínica Las Condes Hospital, Santiago, Chile; 2 Critical Care Department, Faculty of Medicine, Finis Terrae University, Santiago, Chile; 3 Hospital de Urgencia Asistencia Pública, Santiago, Chile; 4 Multidisciplinary Sepsis Unit, Hospital Son Llàtzer, Palma, Spain; 5 Multidisciplinary Sepsis Group, Health Research Institute of the Balearic Islands (IdISBa), Palma, Spain; 6 Intensive Care Unit, Hospital Juaneda Miramar, Palma, Spain; 7 Neurology Department, Clínica Las Condes Hospital, Santiago, Chile; 8 Critical Care Department, Centro de Pacientes Críticos, Clínica Las Condes and Finis Terrae University, Estoril 450, Las Condes, Santiago, Chile

**Keywords:** Brain injuries, Traumatic, Glial fibrillary acidic protein, Ubiquitin thiolesterase, Biomarkers, Clinical decision rules, Multidetector computed tomography, Emergency service, Hospital, Unnecessary procedures

## Abstract

**Background:**

Mild traumatic brain injury (mTBI) accounts for 80% of TBI cases. Although only 16% show intracranial lesions and <1% require neurosurgical intervention, CT overutilization remains common. Clinical decision rules like the Canadian CT Head Rule achieve high sensitivity (≈100%) but poor specificity (28-65%). Serum biomarkers, particularly GFAP and UCH-L1, offer complementary diagnostic performance. Emerging evidence suggests combining both strategies may optimize diagnostic accuracy, though integrated approaches remain poorly characterized in the literature.

**Methods:**

We conducted a narrative review of literature published January 2000-March 2025 across PubMed/MEDLINE, Google Scholar, and Scielo. Search terms included mTBI, biomarkers (GFAP, UCH-L1, S100B), clinical decision rules (Canadian CT Head Rule, New Orleans Criteria, NEXUS), and resource-limited settings. Inclusion criteria comprised observational cohorts, clinical trials, validation studies, systematic reviews, and meta-analyses focused on mTBI in emergency contexts.

**Results:**

Combined strategies integrating clinical decision rules with biomarkers achieved superior diagnostic performance compared to either tool individually. The Canadian CT Head Rule demonstrated optimal performance across GCS 13-15 (sensitivity 93-100% and specificity 28-65% for intracranial lesions). GFAP demonstrated superior diagnostic performance compared with UCH-L1 and S100B. Although UCH-L1 did not provide meaningful incremental value beyond GFAP alone, all currently FDA- and CE-cleared platforms for clinical use (Abbott i-STAT, Alinity i; bioMérieux VIDAS® TBI) measure both GFAP and UCH-L1 in combination, achieving sensitivities of 95.8–97.3% and specificities of 34.2–41.2%.

**Conclusion:**

Integrating the Canadian CT Head Rule with GFAP-based biomarker testing may optimize CT utilization in mTBI. We propose a sequential diagnostic algorithm consisting of initial evaluation with the Canadian CT Head Rule, followed by biomarker testing in CCHR-positive cases, with CT reserved for biomarker-positive patients. This stepwise approach has the potential to support more efficient referral decisions and resource utilization in settings with limited access to neuroimaging, while reducing unnecessary brain CT use in centers with imaging availability, adapting to diverse healthcare contexts. Cost-effectiveness analyses and validation in resource-limited environments remain needed.

## Introduction

Mild traumatic brain injury (mTBI) is commonly defined as cranial trauma that causes loss of consciousness, amnesia, or disorientation, accompanied by a Glasgow Coma Scale (GCS) score of 13–15 [1], although no universally accepted definition exists and considerable heterogeneity persists across clinical practice guidelines and research studies [2]. *See Methodology for details.* It represents approximately 80% of all TBI cases—mild, moderate, and severe— [3, 4]. The worldwide incidence in 2019 was estimated at 12.3 million cases, contributing to a global burden of 1.37 million Years Lived with Disability (YLDs) [5]. Additionally, it generates high impact on health systems, with series reporting hospital admission rates as high as 48% [6], and among hospitalized patients, up to 44.7% requiring intensive care units [7]. Although mortality rates are relatively low (0.1–2.4%) [8, 9], long-term sequelae are substantial. Post-concussive symptoms occur in 16–31% of patients at 3–6 months, and functional disability affects 29–54% [10], with additional increased risk of psychiatric disorders such as depression and post-traumatic stress disorder [11, 12].

Despite this substantial healthcare utilization, the clinical reality reveals that mTBI infrequently presents acute complications requiring urgent management. Only 16% of patients with mTBI show intracranial lesions on computed tomography (CT) [13], and less than 1% require neurosurgical intervention: In one large cohort of 1,664 patients, 0.3% required interventions such as endotracheal intubation, administration of anticonvulsants or anti-edema therapy, or neurosurgery. Among these, only one patient (0.06%) required a craniotomy as a surgical procedure [14]. Even among the subset of patients with confirmed intracranial lesions, only 7–10% ultimately require a neurosurgical procedure [8, 15], most commonly craniotomy or placement of intracranial pressure monitoring devices. This low but real risk of intracranial injury frequently leads to unnecessary imaging studies, most of which yield negative results.

Consequently, brain CT, considered the gold standard for detecting acute traumatic intracranial lesions [16, 17], faces two significant challenges. First, access limitations that restrict its availability in low-complexity medical care services in rural and urban areas, as occur in low- and middle-income regions such as Latin America (LATAM) [18], forcing potentially avoidable referral of these patients to tertiary care level centers. Second, in centers where CT is available, the lack of strategies to optimize diagnostic performance generates overuse in emergency departments [19], increasing costs [20] and prolonging length of stay [21, 22].

This overutilization becomes particularly relevant when considering that less than 1% of all patients consulting the emergency department for mTBI will require neurosurgical intervention [14]. Furthermore, unnecessary CT utilization unnecessarily exposes patients to ionizing radiation, with the consequent carcinogenic risk [23]. Both problems —lack of access in some contexts and overutilization in others— highlight the urgent need to develop strategies that optimize the rational use of brain CT in patients with mTBI, maximizing its diagnostic benefit while minimizing both access barriers and unnecessary resource use.

In response to this scenario, different strategies have been developed to optimize the rational use of brain CT in patients with mTBI. On one hand, clinical decision rules —such as the Canadian CT Head Rule (CCHR)— have been established as validated tools for optimizing neuroimaging decisions in this context [2]. However, while these rules demonstrate sensitivity close to 100%, their limited specificity does not significantly reduce unnecessary brain CT scans in mTBI patients [24, 25]. Alternatively, serum biomarkers of brain damage —such as glial fibrillary acidic protein (GFAP) and ubiquitin C-terminal hydrolase (UCH-L1)— have emerged as promising tools for predicting CT-detectable intracranial lesions [26]. Recent FDA- and CE-cleared GFAP/UCH-L1 platforms underscore the growing clinical feasibility of blood-based biomarkers for mTBI [27]. These biological markers offer particular advantages for both identified problems: they can be implemented in low-complexity healthcare settings with limited access to imaging studies through Point-of-Care (POC) tests [28], and simultaneously contribute to reducing overutilization in centers with imaging capabilities. The integration of clinical decision rules and serum biomarkers represents a promising strategy for optimizing CT utilization. This combined approach could simultaneously improve access in resource-limited environments while reducing unnecessary imaging in well-equipped facilities.

Several recent comprehensive reviews have addressed traumatic brain injury (TBI) biomarkers from different perspectives. Kim et al. (2018) focused on mTBI biomarkers primarily in student-athlete populations, examining correlations with post-concussive symptoms but without addressing applications in the emergency department or integration with clinical decision tools [29]. Wang et al. (2018) provided an extensive review of brain injury biomarkers categorized by temporal profile (acute, subacute, and chronic phases) and identified critical knowledge gaps regarding their utility for rule-out strategies in emergency settings [30]. Ghaith et al. (2022) offered a broad overview of biomarkers across the full TBI severity spectrum, with particular emphasis on predicting post-concussive symptoms and chronic traumatic encephalopathy [31]. While these reviews have substantially advanced the understanding of TBI biomarkers, none have systematically examined their integration with established clinical decision rules in emergency care, nor have they addressed practical implementation strategies for optimizing resource utilization, particularly in resource-limited settings.

This narrative review aims to synthesize the available evidence on diagnostic strategies to optimize brain CT use in adult patients with mTBI. Specifically, it analyzes the diagnostic performance and limitations of established clinical decision rules (CCHR, NOC, NEXUS), evaluates the role and availability of serum biomarkers (GFAP, UCH-L1) as complementary tools, examines evidence on their combined use as an integrated strategy, and determines which of these approaches offers the best solution to resolve the identified problems of limited access and neuroimaging overutilization.

## Methodology

### Study design

A structured search of scientific literature published between January 1, 2000 and March 31, 2025 was conducted in PubMed/MEDLINE, Google Scholar and Scielo databases. The latter was specifically added to incorporate Latin American studies considering the relevance of low-resource contexts. Spanish language terms were included to ensure comprehensive capture of Latin American research.

### Search strategy

Combinations of the following terms were used in English and Spanish: “mild traumatic brain injury” / “traumatismo craneoencefálico leve”, “TBI” / “TEC” / “TCE”, “mTBI”, “biomarkers” / “biomarcadores”, “GFAP”, “UCH-L1”, “S100B”, “clinical decision rules” / “reglas clínicas de decisión”, “Canadian CT-head Rule”, “New Orleans Criteria”, “NEXUS”, “emergency department” / “servicio de urgencias”, “limited resources” / “recursos limitados” and “Latin America” / “Latinoamérica”.

### Selection criteria

Inclusion criteria comprised articles focusing on mTBI across different GCS definitions (13–15 range) in emergency contexts, including observational cohort studies, controlled clinical trials, clinical rules and biomarker validation studies, systematic reviews and meta-analyses. Only articles published in English and Spanish were considered.

Exclusion criteria included individual case reports, case series, abstracts without full article available, studies focused exclusively on moderate or severe TBI, and articles without full text access after author request. The selection process was performed by two independent reviewers through initial review of titles and abstracts to identify potentially relevant articles, followed by full text review of preselected articles. A third reviewer served as arbiter in cases of disagreement regarding article inclusion.

### Study analysis

Given the narrative nature of this review, no formal quality assessment of individual articles was performed, as the objective was to conduct a qualitative analysis of published evidence and synthesize the diversity of results rather than generate summary metrics or quantitative synthesis. A wide diversity of studies was included to capture comprehensive findings across different methodological approaches. Conceptual integration of findings was prioritized over presentation of individual metrics. Relevant findings from each article were presented through tables for comparison and interpretation, focusing on diagnostic performance and clinical applicability of the evaluated tools.

### mTBI definition and study inclusion approach

**Fig. 1:**
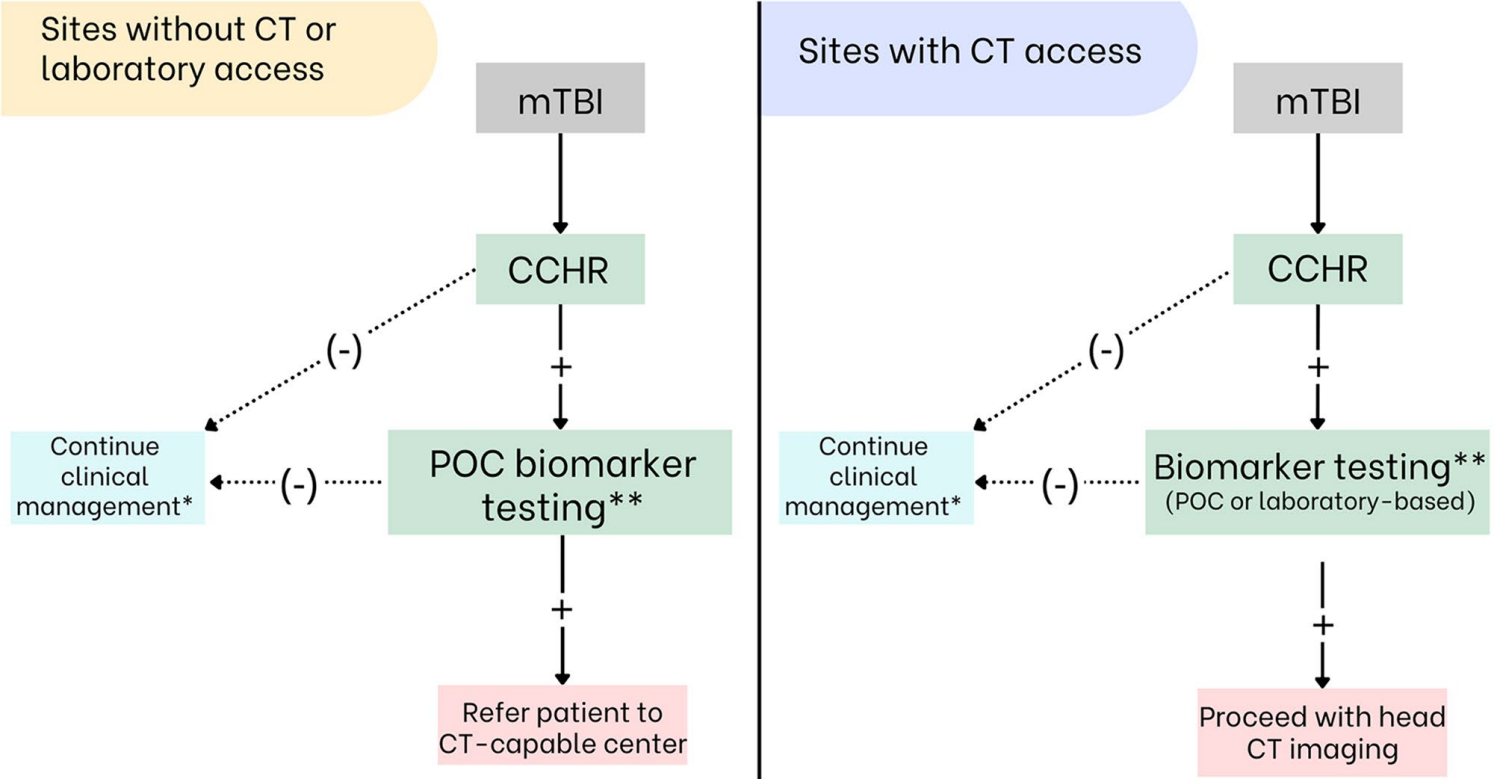
Proposed algorithm for sequential diagnostic strategy in scenarios with and without CT access. mTBI: blunt head trauma followed by signs of neurological dysfunction (loss of consciousness, amnesia, or disorientation) and GCS 13–15, CCHR: Canadian CT-Head Rule, CT: computed tomography, POC: point of care. * No further evaluation for intracranial injury is required; routine clinical care may continue. ** Use of any GFAP/UCH-L1 assay with formal regulatory clearance (FDA or CE). Testing must be performed according to the manufacturer’s validated instructions for use for the specific platform

No universally accepted definition of mTBI exists, and considerable heterogeneity persists across clinical practice guidelines and research studies regarding GCS cutoffs (13–15 vs. 14–15 vs. 15 only), the role of loss of consciousness or amnesia as required criteria, and whether intracranial lesions on imaging preclude an mTBI classification [2]. To provide comprehensive evidence synthesis, this narrative review included studies employing various mTBI definitions. When studies restricted enrollment to specific GCS subgroups, this is explicitly noted in the corresponding sections. Tables 2 and 3 stratify clinical decision rule performance by GCS category (13–15 vs. 15 only) to facilitate appropriate clinical application. For our proposed diagnostic algorithm (Fig. 1), we define mTBI as blunt head trauma followed by signs of neurological dysfunction (loss of consciousness, amnesia, or disorientation) and GCS 13–15, consistent with the most widely used definition in the literature.

## Utility of clinical rules

Various clinical prediction rules have been developed and validated to optimize computed tomography indication in patients with mTBI. Table 1 shows the clinical variables of the main decision rules mentioned in the literature.

The Canadian CT Head Rule (CCHR), developed for patients with GCS 13–15, initially demonstrated a sensitivity of 98.4% and specificity of 49.5% for detecting acute traumatic intracranial lesions, and achieved 100% sensitivity with 66.7% specificity for predicting neurosurgical intervention [24]. Subsequent validation studies confirmed the 100% sensitivity but showed reduced specificity (28.2%) for intracranial lesion detection [25].

The New Orleans Criteria (NOC) was designed specifically for TBI patients with GCS 15. The original study showed 100% sensitivity and 25% specificity for detecting brain CT lesions [32]. External validation revealed suboptimal performance, with both lower specificity (12.7%) and reduced sensitivity (86%) for clinical rule-out purposes [33, 34].

The National Emergency X-Radiography Utilization Study (NEXUS) represents another clinical decision rule for head trauma patients. The derivation study included all patients with blunt head trauma undergoing brain CT regardless of injury severity (mild, moderate, and severe TBI), demonstrating 98.3% sensitivity and 13.7% specificity overall. Among patients with GCS 15, sensitivity was 95.2% with 17.3% specificity for detecting intracranial lesions [35]. Subsequent validation showed improved performance (99% sensitivity, 25.6% specificity) but again included patients across all trauma severities without GCS-based stratification [36].

Available evidence shows that CCHR has been predominantly evaluated across the complete mTBI spectrum (GCS 13–15) (Table 2), while NOC and NEXUS have been validated primarily in patients with GCS 15, thus limiting their applicability to the GCS 13–14 subgroup.

**Table 1:** Clinical decision rules

	Canadian CT Head Rule (24)	NEXUS Head CT (35)	New Orleans Criteria (32)
High-risk criteria	**Any of**: • GCS < 15 > 2 h post-injury • Suspected open skull fracture • Signs of basal skull fracture • > 1 episode of vomiting • Age > 64 years	**Any of**: • Physical evidence of skull fracture • Scalp hematoma • Neurological deficit • Abnormal level of alertness • Abnormal behavior • Persistent vomiting • Coagulopathy • Age ≥ 65 years	**Any of**: • Headache • Vomiting • Age > 60 years • Drug or alcohol intoxication • Short-term memory deficits • Physical evidence of trauma above clavicular level • Post-traumatic seizure
Exclusion criteria	• Age < 16 years • Anticoagulant use • Post-injury seizure	(-)	• GCS < 15 • Age ≤ 3 años

GCS: Glasgow Coma Scale

Considering the low prevalence of CT-detectable intracranial lesions in mTBI patients and the even lower rates of neurosurgical intervention, these clinical decision rules serve as tools to optimize patient selection for neuroimaging [24, 25, 32–35]. Their primary utility lies in ruling out intracranial lesions when negative, potentially eliminating the need for brain CT. This rule-out capability is supported by high sensitivity and favorable negative predictive values (NPV) and negative likelihood ratios (LR(-)). However, poor specificity has limited their effectiveness in reducing brain CT overuse (Tables 2 and 3).

Comparative analysis of the three clinical scales reveals CCHR as having the greatest clinical utility in mTBI patients. This superiority is based on several factors. First, CCHR consistently demonstrates high sensitivity for intracranial lesions (93–100%) and neurosurgical intervention (100%) across the entire mTBI spectrum (GCS 13–15). Second, while its specificity is moderate (28–65%), this represents an acceptable trade-off when prioritizing sensitivity to avoid missing significant lesions. Additionally, its consistently low LR(-) values reinforce its advantages over other rules, even when compared to GCS 15-only subgroups. Importantly, Tables 2 and 3 demonstrate that CCHR is the only rule validated across the entire GCS 13–15 range, while NOC and NEXUS are predominantly limited to GCS 15 patients.

An important consideration when evaluating these diagnostic tools is the use of neurosurgical intervention as an outcome measure. This approach introduces potential bias and requires careful interpretation. The literature demonstrates significant heterogeneity in neurosurgical decision-making —particularly for mild to moderate TBI— across neurosurgeons and centers, influenced by local practices, individual preferences, and varied guideline interpretations, even with similar imaging findings [37, 38].

Given this variability, it may be more appropriate to focus these clinical decision rules on predicting intracranial lesions on brain CT rather than surgical intervention. Since therapeutic decisions with identical findings can vary between professionals and institutions, using surgical intervention as a validation parameter introduces systematic bias. Alternative outcomes such as hospital admission or return visits for persistent symptoms may provide more consistent and objective reference standards.

**Table 2:** Diagnostic performance of different clinical rules in patients with TBI and GCS 13–15

Study	Clinical Rule	*N*	Intracranial Lesions				Neurosurgical Intervention			
			Sensitivity	Specificity	NPV	LR (-)*	Sensitivity	Specificity	NPV	LR (-)*
Stiell 2001 [24]	CCHR	3,121	98.4% (95% CI, 96–99%)	49.6% (95% CI, 48–51%)	(-)	0.032	100% (95% CI, 92–100%)	68.7% (95% CI, 67–70%)	(-)	0.00
Stiell 2005 [33]	CCHR	2,707	100 (95% CI, 98–100)	41.1 (95% CI, 39–43)	(-)	0.00	100 (95% CI, 91–100)	65.6 (95% CI, 64–67)	(-)	0.00
Papa 2012 [25]	CCHR	431	100% (95% CI, 84–100)	28.2% (95% CI, 24–33)	(-)	0.00	100% (95% CI, 46–100)	66.7% (95% CI, 62–71)	(-)	0.00
Bouida 2013 [34]	CCHR	1,582	95% (95% CI, 92–98%)	65% (95% CI, 62–68%)	99% (95% CI, 98–100%)	0.077	100% (95% CI, 90–100%)	60% (95% CI, 44–76%)	100% (95% CI, 99–100%)	0.00
	NOC	1,582	86% (95% CI, 81–91%)	28% (95% CI, 26–30%)	93% (95% CI, 90–96%)	0.500	82% (95% CI, 69–95%)	26% (95% CI, 24–28%)	99% (95% CI, 98–100%)	0.692

CCHR: Canadian CT head Rule, NOC: New Orleans Criteria

*LR(-) values were not documented in any of the cited articles, they were calculated with the sensitivity and specificity reported in each original study using the formula: LR(-) = (1-Sensitivity)/Specificity

**Table 3:** Diagnostic performance of different clinical rules in patients with TBI and GCS 15

Study	Clinical Rule	*N*	Intracranial Lesions				Neurosurgical Intervention			
			Sensitivity	Specificity	NPV	LR (-)*	Sensitivity	Specificity	NPV	LR (-)*
Stiell 2005 [33]	CCHR	1,822	100 (95% CI, 96–100)	50.6 (95% CI, 48–53)	(-)	0.00	100 (95% CI, 63–100%)	76.3 (95% CI, 74–78%)	(-)	0.00
Bouida 2013 [34]	CCHR	1,249	93% (95% CI, 89–97%)	63% (95% CI, 61–65%)	98% (95% CI, 97–99%)	0.111	100% (95% CI, 86–100%)	58% (95% CI, 55–61%)	100% (95% CI, 99–100%)	0.00
Papa 2012 [25]	CCHR	314	100% (95% CI, 68–100)	35% (95% CI, 30–41)	(-)	0.00	100% (95% CI, 31–100%)	80.7% (95% CI, 76–85%)	(-)	0.00
Stiell 2005 [33]	NOC	1,822	100 (95% CI, 96–100%)	12.7 (95% CI, 11–14%)	(-)	0.00	100 (95% CI, 63–100%)	12.1 (95% CI, 11–14%)	(-)	0.00
Bouida 2013 [34]	NOC	1,249	85% (95% CI, 79–91%)	26% (95% CI, 24–28%)	93% (95% CI, 90–96%)	0.577	96% (95% CI, 88–100%)	26% (95%, CI 23–28%)	99% (95% CI, 98–100%)	0.154
haydell 2000 [28]	NOC	909	100% (95% CI, 95–100%)	25% (95% CI, 22–28%)	100% 95% CI, 99–100%)	0.00	(-)	(-)	(-)	(-)
Papa 2012 [25]	NOC	314	100% (95% CI, 68–100%)	9.9% (95% CI, 7–14%)	(-)	0.00	100% (95% CI, 31–100%)	9.6% (95% CI, 7–14%)	(-)	0.00
Mower 2005 [35]	NEXUS	13,728	95.2% (95.2% CI, 92.2–97.2%)	17.3% (95% CI, 16.5–18%)	99.1% (CI, 98.5–99.5%	0.277	(-)	(-)	(-)	(-)

CCHR: Canadian CT head Rule, NEXUS: National Emergency X-Radiography Utilization Study, NOC: New Orleans Criteria

*LR(-) values were not documented in any of the cited articles, they were calculated with the sensitivity and specificity reported in each original study using the formula: LR(-) = (1-Sensitivity)/Specificity

## Biomarkers and mTBI

Over the past decade, serum biomarkers of brain injury have emerged as valuable complementary diagnostic tools in mTBI evaluation, potentially optimizing CT utilization decisions. These proteins are released into circulation following traumatic injury to brain structures and serve as molecular markers of neuronal and glial damage. However, elevations can also occur in non-traumatic contexts including ischemia, inflammation, non-traumatic subarachnoid hemorrhage, and chronic conditions such as Alzheimer’s disease [39].

Multiple biomarkers have been investigated in the TBI context, with glial fibrillary acidic protein (GFAP), ubiquitin C-terminal hydrolase-L1 (UCH-L1), and S100B protein showing particularly promising diagnostic performance [31]. Several studies have reported correlations between plasma biomarker levels and acute traumatic intracranial lesions on brain CT [26, 40–43]. Their diagnostic characteristics and performance metrics are detailed in Tables 4 and 5, and 6.

**Table 4:** Comparative table of generalities of different biomarkers useful in mTBI

Biomarker	Cellular Origin	Diagnostic window	Limitations
GFAP	Astroglial cells	Detectable in 60 min Peak at 20 h Detectable up to 7 days	Limited availability in LATAM
UCH-L1	Neuronal soma	Faster initial elevation than GFAP Detectable within 60 min Peak at 8 h Detectable for 48 h	Limited availability in LATAM
S100B	Astroglial cells; adipocytes, bone marrow, muscle cells	Immediate elevation Half-life up to 120 min	False positives due to release from other tissues Short half-life

Adapted from Biberthaler et al. [40], and Papa et al. [43]

**Table 5:** Regulatory-cleared GFAP/UCH-L1 platforms and key analytical and operational characteristics

Platform / Manufacturer	Sample type	Assay technology	Time window	Setting	Regulatory status
Banyan BTI™	Serum or plasma	benchtop chemiluminescent ELISA	≤ 12 h	Not commercially implemented; regulatory predicate only	FDA De Novo, 2018
Abbott i-STAT TBI Plasma Test	EDTA plasma (centrifuged)	microfluidic immunoassay cartridge	≤ 12 h	Clinical laboratory	FDA 510(k), 2021; CE Mark 2021
Abbott i-STAT TBI Whole Blood Test	whole blood (unprocessed)	microfluidic immunoassay cartridge	≤ 24 h	POC	FDA 510(k), 2024
bioMérieux VIDAS^®^ TBI	Serum	ELFA	≤ 12 h	Clinical laboratory	CE Mark 2023; FDA 510(k), 2024
Abbott Alinity i Assay	Serum or plasma	CMIA	≤ 12 h	Clinical laboratory	FDA 510(k), 2023

ELISA: enzyme-linked immunosorbent assay; ELFA: enzyme-linked fluorescent assay; CMIA: chemiluminescent microparticle immunoassay; EDTA: ethylenediaminetetraacetic acid (anticoagulant used in blood collection tubes); POC: point-of-care

**Table 6:** Diagnostic performance of FDA/CE-Cleared GFAP/UCH-L1 platforms

Platform	Supporting cohort	Study size	Cutoff thresholds	Sensitivity	Specificity
Abbott i-STAT TBI Plasma Test	ALERT-TBI [44]	*N* = 1901	GFAP: 30 pg/mL UCH-L1: 360 pg/mL	95.8%	40.4%
Abbott i-STAT TBI Whole Blood Test	Internal FDA 510(k) validation cohort*	*N* = 420	GFAP: 65 pg/mL UCH-L1: 360 pg/mL	96.5%	40.3%
Abbott Alinity i GFAP/UCH-L1 Assay	ALERT-TBI [45]	*N* = 1899	GFAP: 30 pg/mL UCH-L1: 360 pg/mL	96.7%	40.1%
bioMérieux VIDAS^®^ TBI (GFAP, UCH-L1)	ALERT-TBI **	*N* = 1911	GFAP: 22 pg/mL UCH-L1: 327 pg/mL	96.7%	41.2%
	BRAINI ***	*N* = 562	GFAP: 22 pg/mL UCH-L1: 327 pg/mL	97.3%	34.2%

*Clinical performance data for the Abbott i-STAT TBI Whole Blood Test derive from a prospective cohort described exclusively within the FDA 510(k) submission [28], and not from any previously published clinical study

** Performance values attributed to ALERT-TBI correspond to serum samples re-analyzed using the VIDAS^®^ platform as part of the FDA 510(k) submission [69], rather than to results generated in the original ALERT-TBI publication

*** Regulatory performance metrics for the VIDAS^®^ TBI assay correspond to the FDA-reviewed subset of the BRAINI cohort —restricted to serum samples stored ≤ 3.5 years— as detailed in the VIDAS^®^ 510(k) summary [69]. These values differ from those reported in the full published BRAINI cohort [74]

### GFAP and UCH-L1

Glial fibrillary acidic protein (GFAP) is an intermediate filament primarily located in astrocyte cytoskeletons. Beyond its structural role, GFAP serves as an important marker of astrogliosis, the reactive process triggered by brain parenchymal injury [48].

Ubiquitin C-terminal hydrolase L1 (UCH-L1) is a brain-specific enzyme predominantly expressed in neuronal cell bodies, where it plays a critical role in maintaining axonal integrity. This enzyme has been implicated in acute brain injury pathogenesis, including trauma and cerebral ischemia, contributing to white matter preservation and functional recovery [49].

The TRACK-TBI study, a large multicenter cohort with standardized clinical, imaging, and biomarker data, has generated several key publications in the field. In a derivative study including 206 patients across the TBI severity spectrum (83% mild) who presented within 24 h post-injury demonstrated AUC-ROC values of 0.87 (95% CI 0.83–0.90) and 0.91 (95% CI 0.88–0.94) for UCH-L1 and GFAP, respectively. A key finding was that combining both biomarkers improved diagnostic performance beyond either marker individually, achieving an AUC-ROC of 0.94 (95% CI 0.92–0.96) [50].

To evaluate the temporal profile of GFAP and UCH-L1, Papa et al. conducted a study evaluating GFAP and UCH-L1 using blood samples collected at multiple time points from 548 adult trauma patients enrolled within 4 h of injury, all presenting with or without TBI confirmed by CT scan or with a Glasgow Coma Scale score < 15 within the first 24 h post-injury. Although both biomarkers showed elevated levels in patients with TBI, UCH-L1 demonstrated earlier behavior with rapid rises, peaking at approximately 8 h and showing strong diagnostic performance within the first 12 h post-injury. GFAP, in contrast, increases more gradually, peaking at approximately 20 h and remaining detectable for up to 7 days, thus providing diagnostic capability beyond the early window [43]. In another study, Papa et al. evaluated the temporal behavior and diagnostic accuracy of this biomarkers including adult and pediatric population, showing similar results [51]; In both cohorts, GFAP outperformed UCH-L1 in distinguishing TBI from non-TBI trauma and in identifying intracranial injury.

The ALERT-TBI study [26] evaluated the diagnostic performance of GFAP and UCH-L1 in adults with mild traumatic brain injury (GCS 14–15) presenting within 12 h of injury. Using predefined biomarker thresholds (GFAP 10 pg/mL; UCH-L1 80 pg/mL), the combined GFAP + UCH-L1 panel achieved 97.3% sensitivity and 36.7% specificity for detecting acute intracranial lesions on CT. Although the dual-marker strategy outperformed each biomarker individually, its incremental advantage over GFAP alone did not reach statistical significance. Importantly, serum and plasma samples from the ALERT-TBI cohort were subsequently reanalyzed across multiple diagnostic platforms as part of the evidence base for regulatory clearance. For this reason, ALERT-TBI has become one of the most influential and frequently referenced biomarker cohorts in the TBI literature.

Papa et al. [42] later investigated mTBI patients presenting within 4 h of trauma. At established thresholds (GFAP: 67 pg/mL; UCH-L1: 189 pg/mL), GFAP and UCH-L1 individually achieved 87% and 96% sensitivity, and 65% and 29% specificity, respectively. Dual-biomarker analysis yielded 100% sensitivity at the expense of reduced specificity (25%).

In the CENTER-TBI study [52], which included 1,951 mTBI patients presenting within 24 h of injury, GFAP showed superior diagnostic performance compared to S100B and UCH-L1. Additionally, combining GFAP with clinical parameters resulted in further performance improvements.

A systematic review and meta-analysis by Rogan et al. [53] examining biomarker performance within 24 h of mTBI found GFAP sensitivity ranging from 63 to 100% with specificity of 0–89%, while UCH-L1 showed 61–100% sensitivity and 21-63.7% specificity. A subsequent meta-analysis by Amoo et al. [41] analyzed GFAP across 9 studies and, despite significant heterogeneity, identified an optimal threshold of 22 pg/mL yielding 93% sensitivity and 36% specificity.

Notably, several investigations suggest that UCH-L1 fails to provide incremental diagnostic value when added to GFAP alone [26, 41, 42, 52].

### S100B

S100B is the most extensively studied blood biomarker in traumatic brain injury and was the first biomarker implemented clinically to reduce unnecessary head CT scans in mTBI, following its adoption in the 2007 Scandinavian Neurotrauma Committee guidelines [54]. S100B is a calcium-binding protein expressed predominantly in astrocytes, where it modulates intracellular calcium homeostasis [55]. After TBI, blood–brain barrier disruption leads to increased S100B concentrations in both serum and cerebrospinal fluid [56, 57].

However, S100B lacks central nervous system specificity. It is also expressed in adipocytes, melanocytes, chondrocytes, striated and cardiac muscle, and bone marrow [30, 58, 59]. This widespread tissue distribution limits its diagnostic specificity, as extracranial injuries —common in trauma patients— can markedly elevate serum levels and reduce discrimination for intracranial lesions [60, 61]. Additionally, S100B has a short half-life, restricting its clinical utility to a narrow time window—approximately the first three hours after injury [62, 63].

Despite these limitations, S100B has been validated in large clinical cohorts. The landmark study by Biberthaler et al. [40], including 1,309 patients, reported a sensitivity of 99% and specificity of 30% for detecting CT lesions. Systematic reviews have described sensitivity ranging from 63 to 100% and specificity from 5 to 58% [53].

S100B also has been evaluated within the TRACK-TBI study. In a phase 1 biomarker cohort including 1,359 patients across the full TBI severity spectrum, investigators compared serum S100B with plasma GFAP. GFAP demonstrated substantially superior accuracy for detecting CT abnormalities within 24 h showing an AUC-ROC 0.85, compared with 0.67 for S100B [64]. These findings, consistent with prior literature, reinforce GFAP’s superiority over S100B for mTBI evaluation and support the transition toward newer astroglial biomarkers with greater brain specificity.

### MAP-2

Microtubule-associated protein 2 (MAP-2) is a relatively novel neuronal biomarker specifically localized to neuronal cell bodies and dendrites, where it modulates cytoskeletal organization and stability. In a large multicentric trial including patients with moderate to severe TBI, MAP-2 demonstrated considerable diagnostic performance, with an AUC-ROC of 0.78 for detecting intracranial lesions on CT, performing better than UCH-L1 but inferior to GFAP [65]. Although its combination with other biomarkers did not improve diagnostic accuracy beyond GFAP alone, MAP-2 provides valuable complementary information about diffuse injury; however, it does not yet have regulatory approval for clinical use. Unfortunately, large studies focused on MAP-2 diagnostic performance in mTBI are currently lacking.

### Other biomarkers

Beyond acute diagnosis, several additional biomarkers have been investigated to characterize longer-term consequences of mTBI, including post-concussional syndrome and chronic traumatic encephalopathy. Salivary exosomal biomarkers—tau, phosphorylated tau (p-tau), beta amyloid (Aβ), and microRNAs (miRNAs)—represent potential candidates for TBI and post-concussional syndrome assessment [66]. Also, neurofilament light chain (NFL) rise gradually and remain elevated for weeks to months after injury, reflecting ongoing axonal damage rather than acute intracranial pathology [67]. However, these biomarkers, along with neuron-specific enolase (NSE), have shown limited ability to identify patients with acute intracranial lesions who may require urgent neurosurgical intervention or admission for neurocritical care. For this reason, they currently play no role in early triage decisions, where GFAP remains the most clinically informative biomarker [41, 68, 69].

## Regulatory approval biomarker platforms

As presented in Table 5, several GFAP/UCH-L1 diagnostic platforms have now obtained regulatory clearance, supported by clinical evidence from ALERT-TBI, TRACK-TBI, and CENTER-TBI [27]. The first assay to receive regulatory authorization was the Banyan Brain Trauma Indicator™ (BTI) in 2018, cleared through the FDA De Novo pathway. Although it was used in several pivotal studies in the field [26, 42, 43, 50], it was never commercialized due to its long analytical turnaround time; instead, it served as the regulatory predicate for all subsequently cleared platforms [70].

Two automated laboratory-based assays are currently available for clinical use: the Abbott Alinity i GFAP/UCH-L1 assay, cleared by the FDA for serum and plasma testing [71], and the bioMérieux VIDAS^®^ TBI (GFAP, UCH-L1) assay, which received CE marking in 2023 and FDA 510(k) clearance in 2024 [46, 72]. Both platforms require centrifuged samples and dedicated laboratory instrumentation.

In addition, two assays have been developed for the handheld i-STAT device. The first to reach clinical implementation was the Abbott i-STAT TBI plasma test, cleared in 2021 [73, 74]. Despite operating on a portable analyzer, this assay required centrifuged plasma and was explicitly authorized only for clinical laboratory settings, limiting its true point-of-care utility. The more recent Abbott i-STAT TBI whole-blood test, cleared by the FDA in April 2024 [28], measures GFAP and UCH-L1 directly from unprocessed blood, eliminating the need for centrifugation and enabling genuine point-of-care use in settings such as small emergency departments, rural hospitals, and pre-transfer evaluations.

All cleared GFAP/UCH-L1 platforms share the same regulatory indication: they are intended to aid in the evaluation of adults with suspected mild traumatic brain injury (GCS 13–15) within an approved post-injury time window and to support decisions regarding the need for head CT. A negative—or “Not Elevated”—result indicates a very low likelihood of CT-detectable acute intracranial injury. Importantly, across all cleared assays, the test is considered positive if either GFAP or UCH-L1 exceeds its respective regulatory cutoff; only when both biomarkers are below threshold is the result interpreted as “Not Elevated.” For the i-STAT TBI Whole Blood Test, the regulatory indication specified in the FDA clearance additionally requires the presence of at least one clinical feature such as loss of consciousness, amnesia, altered mental status, or a focal neurological deficit.

## Clinical performance of cleared platforms

As summarized in Table 6, the most clinically relevant evidence for GFAP/UCH-L1 testing comes from the datasets submitted to regulatory agencies, where assays were evaluated using predefined cutoffs and standardized endpoints.

The clinical validation of the Abbott i-STAT TBI Plasma Test is based on the ALERT-TBI cohort (*n* = 1901) [44], demonstrating 95.8% sensitivity, 40.4% specificity, and a 99.3% negative predictive value using the FDA-cleared cutoffs for GFAP (30 pg/mL) and UCH-L1 (360 pg/mL). For the Abbott i-STAT TBI Whole Blood Test, the FDA 510(k) submission reports performance derived from a prospective multicenter whole-blood cohort evaluated exclusively within the regulatory dossier, including a 420-patient training set used to establish the cutoff strategy [28]. The Abbott Alinity i assay was similarly validated using serum and plasma samples from ALERT-TBI (*n* = 1899), yielding 96.7% sensitivity and 40.1% specificity at the same regulatory thresholds [45]. For the bioMérieux VIDAS^®^ TBI assay, clearance was supported by two datasets: first, serum samples from ALERT-TBI re-analyzed with the VIDAS^®^ platform (*n* = 1911), which showed 96.7% sensitivity and 41.2% specificity, and second, a restricted subset of the BRAINI cohort (*n* = 562) –consisting of serum samples stored ≤ 3.5 years, yielding 97.3% sensitivity and 34.2% specificity using the CE- and FDA-cleared cutoffs 22 pg/mL for GFAP and 327 pg/mL UCH-L1 [46, 47]. These datasets collectively provide the most pragmatic and clinically actionable estimates of biomarker performance.

## Combined strategies: resource optimization through integration of clinical rules and biomarkers

Emerging research indicates that combining clinical decision rules with biomarkers may further optimize patient selection for neuroimaging [42, 75]. Two key studies have investigated these integrated strategies. Papa et al. [42] evaluated combinations of established clinical rules (CCHR, NOC, NEXUS) with GFAP and UCH-L1 in 349 mTBI patients, finding that CCHR combined with GFAP achieved optimal performance with an AUC-ROC of 0.88 (95% CI: 0.81–0.95). Notably, GFAP significantly enhanced CCHR performance independent of UCH-L1, with the combination outperforming either tool individually and demonstrating clear synergistic effects. Similarly, Undén et al. [76] assessed an algorithm integrating clinical variables with S100B measurement and observed a potential 32% reduction in brain CT overuse. These findings suggest that biomarker-clinical rule integration can effectively optimize mTBI evaluation while reducing unnecessary CT utilization.

## Integrated approach to the diagnostic process: CCHR + POC biomarkers

Based on current evidence, a systematic sequential diagnostic strategy integrating clinical rules and serum biomarkers appears feasible. Initial evaluation using the Canadian CT Head Rule (CCHR), followed by point-of-care (POC) biomarker testing in selected cases, represents a rational approach to optimizing mTBI management. POC implementation could substantially reduce logistical and infrastructure barriers, making this strategy viable even in resource-limited settings. This integrated approach leverages the complementary strengths of both tools: CCHR’s high sensitivity for detecting cases requiring intervention and the combined evaluation’s capacity to reduce unnecessary referrals and imaging studies. Therefore, we propose implementation algorithms for scenarios with and without CT availability, while maintaining optimal patient safety (Fig. 1).

## Special situations: implications of alcohol intake

Concurrent alcohol consumption is common in TBI patients, with prevalence ranging from 37% to 51% [77]. Alcohol intoxication can impair consciousness assessment and mask neurological signs, complicating the application of clinical rules that rely on neurological evaluation. Although CT positivity among intoxicated patients has been reported as low as 1.9% [78], a secondary analysis of the TRACK-TBI study [79] investigated the effect of elevated blood alcohol levels on the diagnostic accuracy of GFAP and UCH-L1 across the full spectrum of TBI severity. This cohort of 2,320 TBI patients showed that patients with markedly elevated blood alcohol concentrations had higher rates of CT-detectable intracranial lesions. Importantly, the performance of individual biomarkers differed: GFAP demonstrated substantially higher diagnostic accuracy in patients with significantly elevated alcohol levels (AUC-ROC = 0.954) compared with those with lower or no elevation (AUC-ROC 0.849–0.879), whereas UCH-L1 showed no significant performance variation across alcohol categories (AUC-ROC 0.657–0.733). Consequently, the combined GFAP + UCH-L1 model also performed better among patients with high alcohol levels, driven primarily by GFAP (AUC-ROC = 0.949) [79]. These findings indicate that alcohol does not impair —and in the case of GFAP may enhance— biomarker diagnostic discriminability in mTBI evaluation.

## TBI in low to middle income countries (LMIC) and biomarkers

Traumatic brain injury (TBI) represents a disproportionately high burden in low- and middle-income countries (LMICs), where both incidence and mortality are reported to be three times higher compared with high-income nations [80]. A recent review has outlined key epidemiological data from these settings, showing a predominance of young male patients (second and third decades of life), with substantial regional variation: male-to-female ratios of 3–24:1 in Africa, 1.5–10.4:1 in Asia, and 4.3–6.7:1 in South America. Moreover, important differences have been documented regarding adherence to recommended interventions outlined in international guideline standards [81].

Despite the limited number of studies addressing traumatic brain injury in LMICs—many of which are reference articles published more than a decade ago [82]—their findings consistently show that TBI in these populations behaves similarly to that observed in other regions, with older age and lower Glasgow Coma Scale scores being strongly correlated with the risk of intracranial hemorrhagic lesions. However, meaningful differences exist in prognostic implications, particularly for severe TBI, which carries a mortality rate estimated to be 2–3 times higher than that reported in high-income countries [83].

In searching the literature using the terms “Latin America” and searching for articles in Spanish, we did not find any articles that met these criteria and contributed to the objective of this review.

From the authors’ perspective, although the use of biomarkers in TBI remains underrepresented in low- and middle-income countries, and despite the absence of data to definitively support the hypothesis of equivalent diagnostic performance in this specific subpopulation, it is reasonable to postulate that, given the epidemiological similarities of TBI in terms of gender distribution, age, and underlying mechanisms across different socioeconomic contexts, biomarkers could represent a relatively accessible implementation strategy to reduce the gap in clinical outcomes and TBI impact between low- and middle-income countries and high-income countries. This hypothesis gains particular relevance considering that, despite shared epidemiological characteristics, significant differences in mortality and outcomes persist, suggesting that disparities do not lie in the intrinsic nature of the injury, but rather in the available diagnostic and therapeutic capabilities.

## Potential uses and knowledge gaps

The combined use of these biomarkers with clinical decision rules such as the Canadian CT Head Rule (CCHR) shows potential utility in mTBI hospital management by enabling timely and appropriate brain CT ordering decisions, thereby reducing healthcare costs, emergency department length of stay, and the need for specialist consultations (neuroradiology, neurology, neurosurgery). However, limited literature specifically examines whether this integrated approach actually reduces emergency department length of stay, patient boarding times, patient satisfaction, and other hospital management metrics.

Point-of-care (POC) systems—portable analytical devices used directly at the bedside—offer a simple tool alternative for biomarker measurement. When used appropriately in conjunction with established clinical decision rules, these systems can effectively reduce unnecessary referrals from primary care facilities to higher-complexity centers.

Standardized referral protocols between facilities with different complexity levels should incorporate clear criteria based on validated clinical decision rules and biomarker testing. This approach would optimize communication across different care levels. Outcome monitoring through patient registries could enable evaluation of the real-world impact of implementing this integrated strategy in diverse healthcare settings, facilitating protocol adjustments based on local experience and outcomes.

## Concluding remarks

mTBI represents a frequent diagnostic challenge in emergency departments, particularly in resource-limited settings. This review has analyzed the diagnostic performance of clinical decision rules such as the CCHR and serum biomarkers including GFAP and UCH-L1, showing that combining both approaches yields superior diagnostic performance compared to either tool used individually. Notably, several investigations suggest that UCH-L1 does not provide incremental diagnostic value when added to GFAP alone, indicating that GFAP may be the primary biomarker of clinical utility. The recent availability of point-of-care systems for biomarker testing facilitates implementation of stepwise diagnostic strategies across diverse healthcare settings.

Implementation of integrated diagnostic algorithms combining the CCHR with biomarkers would provide multiple benefits tailored to different resource levels: in settings with limited CT access, this approach could reduce unnecessary referrals and optimize healthcare transport resources; in centers with greater resource availability, it could mitigate the adverse effects of CT overutilization, including excessive healthcare costs, prolonged emergency department stays, and unnecessary radiation exposure. Special clinical scenarios such as alcohol intoxication represent situations where these strategies offer additional advantages, maintaining diagnostic utility when clinical assessment may be compromised.

While important research opportunities remain, including targeted studies in resource-limited environments and cost-effectiveness analyses adapted to different socioeconomic contexts, integrating clinical decision rules with biomarkers represents a significant advance toward more efficient and equitable diagnostic approaches in mTBI management. Precision medicine should not be confined to resource-rich environments; the true challenge lies in strategically adapting and implementing these tools so that all patients can benefit from their potential, regardless of their healthcare setting.

## Data Availability

This narrative review is based on publicly available literature cited in the references.

## Abbreviations

CCHR: Canadian CT head Rule
CT: Computed tomography
GFAP: Glial fibrillary acidic protein
GCS: Glasgow Coma Scale
LATAM: Latin America
LR(-): Negative likelihood ratio
mTBI: Mild traumatic brain injury
NEXUS: *National Emergency X-Radiography Utilization Study*
NOC: New Orleans Criteria
NPV: Negative predictive value
POC: Point-of-care
TBI: Traumatic brain injury
UCH-L1: Ubiquitin C-terminal hydrolase L1
YLDs: Years Lived with Disability
